# Plasticity in the structure and assembly of proteasomes

**DOI:** 10.1016/j.jbc.2026.111334

**Published:** 2026-03-02

**Authors:** Alana H. Chang, Swarnab Sengupta, Robert J. Tomko

**Affiliations:** Department of Biomedical Sciences, Florida State University College of Medicine, Tallahassee, Florida, USA

**Keywords:** proteasome, ubiquitin, macromolecular assembly, ATPase, proteolysis, protease

## Abstract

Proteasomes are large multisubunit protease complexes found in all domains of life, where they execute regulatory and quality control degradation critical for organismal health. The canonical form of the proteasome, known as the 26S proteasome, consists of a 28-subunit barrel-shaped proteolytic core particle (CP) that is capped on its barrel ends by the 19-subunit regulatory particle (RP). The RP recognizes and captures substrates destined for degradation, mechanically unfolds them using energy derived from ATP, and translocates them through a gated pore at the surface of the CP into the proteolytic sites housed in its hollow center. Due to their exceptional size and subunit complexity, biogenesis of 26S proteasomes is a highly orchestrated process facilitated by dedicated assembly chaperones and conserved features of its subunits. Since the initial discovery of canonical 26S proteasomes, numerous noncanonical CPs harboring distinct subunit compositions have been detected, as have several alternative non-RP regulators of CP function. Here, we review the structure and assembly of canonical and noncanonical forms of the proteasome and highlight recent structural studies that have greatly clarified our understanding of how these fascinating and complicated molecular machines form rapidly and faithfully in the cellular milieu. In addition, we explore the assembly mechanisms that yield plasticity in the subunit composition of proteasomes, as well as emerging evidence of plasticity in the assembly pathways by which proteasomes are built in cells.

All domains of life rely on regulated proteolysis to destroy toxic misfolded proteins, remove temporally acting regulatory proteins, and maintain the quality of the proteome. In eukaryotes, much of this regulatory protein degradation is mediated by the 26S proteasome, a 66-subunit ATP-dependent multicatalytic peptidase complex. Whereas other proteolytic systems often display rather indiscriminate substrate preference, the 26S proteasome can selectively destroy individual protein molecules with exquisite precision by coupling degradation to the modification of the substrate with chains of the small protein ubiquitin. Since the initial discovery of the 26S proteasome and its linkage to ATP- and ubiquitin-dependent degradation in the late 1970s and 80s (1, 2, 3, 4, 5, 6, 7, 8, 9), it has emerged as an invaluable model for understanding mechanochemical coupling, coordination of activities within multienzyme complexes, and assembly of complex macromolecular structures in cells.

In the past 3 decades, contributions from laboratories spanning the globe have revealed an unexpected diversity of noncanonical proteasomes in eukaryotes. Each such discovery has revealed new biology with important implications for health and disease. In this review, we perform a “census” of proteasome variants in eukaryotes and explore how canonical and noncanonical proteasomes are assembled from their individual subunit building blocks. To facilitate discussion, we will first briefly review the structure and function of the canonical 26S proteasome, which has been addressed in detail in several outstanding recent reviews (10, 11, 12). We will then examine the compositional diversity of known eukaryotic proteasomes. Finally, we review recent advances in knowledge of proteasome biogenesis driven by advances in the structural biology of assembly intermediates, and explore plasticity in the mechanisms and pathways that build these fascinating molecular machines. For simplicity and clarity, we will use the unified proteasome subunit nomenclature (13) for proteasome subunits and the common mammalian names for all other proteins discussed.

## The 26S proteasome

**Figure 1:**
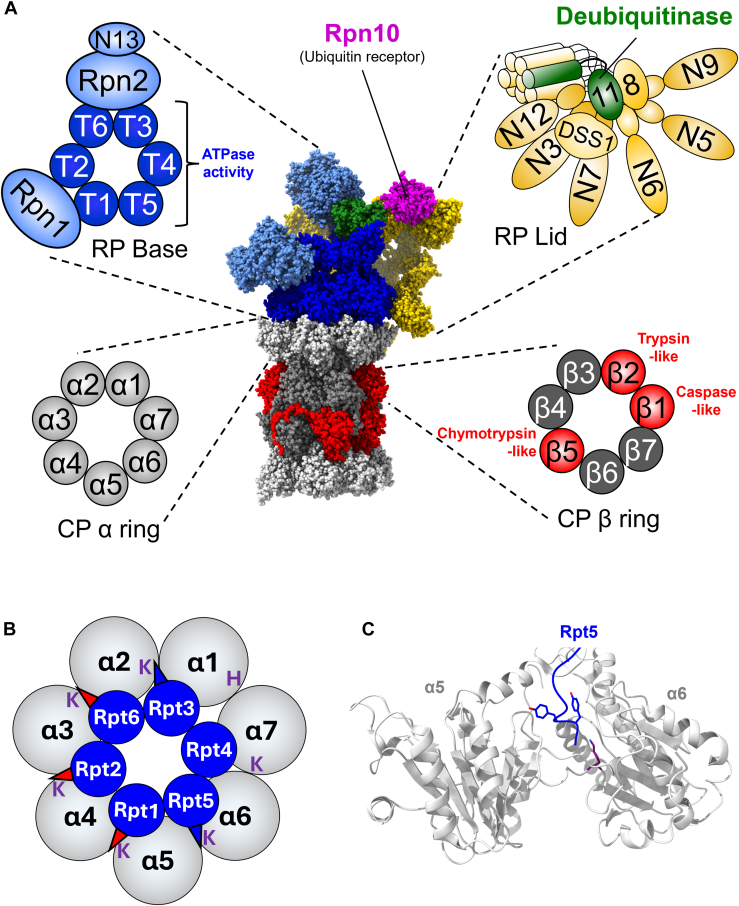
**Subunit composition and ultrastructure of the canonical 26S proteasome.***A*, the structure of the human canonical 26S proteasome is shown (Protein Data Bank ID = 5T0C), with an expanded view of key structural/functional elements in *cartoon representation*. 20S core particle (CP) α rings are shown in *light gray*, noncatalytic β subunits in *dark gray*, and catalytic β subunits in *red*. The regulatory particle (RP) base is shown with ATPase subunits Rpt1–6 in *dark blue* and non-ATPase base subunits Rpn1, Rpn2, and Rpn13 in *light blue*. RP lid subunits are shown in *gold*, with the deubiquitinase subunit Rpn11 shown in *green*. The major ubiquitin receptor Rpn10 is shown in *magenta*. *B*, the arrangement of the 19S RP base ATPase ring upon the surface of the α ring is shown. Rpt and α subunits are not shown to scale. The C termini of ATPase subunits that are stably docked into their cognate α ring pockets in the human 26S proteasome are shown as *blue triangles*. Those that undergo docking upon activation of the proteasome for proteolysis are shown as *red triangles*. The α pocket lysine residues with which the C termini interact are shown in *purple font*. Note that the α1–α7 pocket uniquely holds a nonlysine residue at this position, with histidine being present in most higher eukaryotes. In yeast 26S proteasomes, the Rpt2 C terminus appears stably docked in the α3–α4 pocket in available cryo-EM structures. *C*, representative interaction of a 19S RP base ATPase C terminus with the α ring pocket. The C-terminal HbYX motif of Rpt5 (*blue*) is shown in *stick mode*, with Lys62 of α6 shown in *purple stick mode*.

The canonical 26S proteasome is present in all eukaryotes and is the largest and most complex form of the proteasome. With 20 distinct enzymatic centers and a mass of ∼2.5 MDa, it rivals the size and complexity of the ribosome. Like all proteasomes, it can be biochemically divided into a 20S core particle (CP) that harbors the proteolytic centers and regulatory “caps” that dock onto one or both barrel ends of the CP (Fig. 1*A*). The regulatory caps regulate substrate access to the CP, and in some cases, preprocess substrates destined for proteolytic cleavage. For the canonical 26S proteasome, this cap is called the 19S regulatory particle (RP).

The CP of the canonical 26S proteasome consists of four stacked heteroheptameric α and β rings, each containing seven distinct α (α1–α7) or β (β1–β7) subunits. The β rings are sandwiched between two α rings, which interface with CP regulators. The β1, β2, and β5 subunits harbor caspase-like, tryptic-like, and chymotryptic-like activities, respectively. The 19S RP can be biochemically separated into base and lid subcomplexes (14). The base contains a heterohexameric ring of AAA+ family ATPase subunits, Rpt1–6, that act as the ATP-dependent motor of the 26S proteasome (10, 15). The Rpt ATPase ring is adorned with three non-ATPase subunits, Rpn1, Rpn2, and Rpn13, which serve as substrate receptors and as docking sites for accessory proteins that fine-tune the activities of the 26S proteasome. The lid contains nine subunits: Rpn3, Rpn5–9, Rpn11, Rpn12, and DSS1 (Sem1 in yeast). Rpn11 is the sole intrinsic subunit capable of removing the polyubiquitin degradation signal from substrates during their degradation. In addition to deubiquitination, the lid serves in part to transmit conformational changes during substrate processing and degradation within the proteasome that coordinate the enzymatic activities of the catalytic subunits within the CP and RP (16, 17).

Elegant work from multiple laboratories has recently clarified the mechanism of substrate degradation by canonical 26S proteasomes: engagement of an unstructured region of a captured substrate by the ATPase pore promotes a conformational rearrangement in the RP that triggers ATP-dependent unfolding of the substrate. This rearrangement is associated with the opening of a proteinaceous gate into the peptidase chamber composed of several α-subunit N termini (18). Gate opening is triggered by the insertion of selected RP ATPase subunit C termini, several of which contain so-called HbYX motifs (where Hb is hydrophobic, Y is tyrosine, and X is any amino acid), into pockets formed at the interfaces of adjacent α subunits (Fig. 1*B*) (19, 20, 21, 22, 23, 24, 25). Cryo-EM structures of open-gate 26S proteasomes display densities corresponding to the Rpt3, Rpt2, and Rpt5 HbYX motifs docked in the α1–α2, α3–α4, and α5–α6 pockets, respectively. Rpt6 and Rpt1 HbYX-like motifs are also observed docked in the α2–α3 and α4–α5 pockets (19, 20) (Fig. 1*B*), suggesting these five termini may jointly be required for gate opening. In these α subunit pockets, the HbYX motif C-terminal carboxylate hydrogen bonds with the ε-amino group of a conserved lysine present in the counterclockwise α subunit, with the tyrosine side-chain hydrogen bonding with conserved residues in α subunit N termini to help open and stabilize the gate (Fig. 1*C*). Substrate unfolding by the ATPases drives both the translocation of the unfolded portion of the substrate into the CP peptidase sites as well as the cotranslocational removal of the polyubiquitin targeting signal by Rpn11 (26, 27, 28).

## Diversity among CPs and regulators

Over the past few decades, a flurry of discoveries reporting alternative regulators, CPs harboring noncanonical subunits, and CPs with noncanonical arrangements of canonical subunits has occurred. This increased wealth of subunits, subunit arrangements, and regulators could theoretically lead to a staggering number of proteasome variants, each conferring unique properties to substrate recognition, processing, or proteolytic product generation. Notably, distinct proteasome variants select different subsets of substrates and yield distinct peptide products (29, 30), accentuating the underappreciated role of the proteasome as a key specificity factor in protein degradation. Understanding and deconvoluting the activities of alternative proteasomes is especially crucial given recent work showing that the peptide products of proteasomal degradation have important biological activities, such as neuronal signaling (27, 28), adaptive immunity (31), and microbial defense (32). Below, we delineate known forms of the proteasome present in bacteria, archaea, and eukaryotes and speculate on the breadth of distinct proteasome variants that may exist in humans.

### Plasticity in the composition of the 20S CP

20S CPs likely evolved from a monomeric N-terminal hydrolase protein already existing in the last universal common ancestor (33). Although CPs are present in all eukaryotes and archaea examined to date, genes encoding bacterial CPs appear limited primarily to the *Actinomycetales* and *Nitrospirales* orders (34) and have potentially been lost in many bacterial lineages (33). With the apparent sole exception of *Rhodococcus erythropolis*, all species within these orders encode a single α and β subunit, yielding a single form of the 20S CP with homoheptameric α and β rings. Although *R. erythropolis* encodes two α and two β subunits, it is unclear if they cohabitate individual CPs or if they form distinct homogenous or heterogenous CPs (35). In contrast to bacteria, archaea often encode two to three distinct α or β subunits (36), permitting the production of distinct CPs with homomeric rings as well as CPs harboring heteromeric α or β rings. Further, CPs harboring two distinct homomeric α rings have also been reported (37). Most evidence to date indicates that archaea primarily produce a single form of CP, but in response to changes in environmental conditions, will enhance the production of alternative subunits to yield alternate CPs with mixtures of α or β subunits (37, 38). In most cases, how this alters proteolysis or otherwise benefits the microbe is unknown.

**Figure 2:**
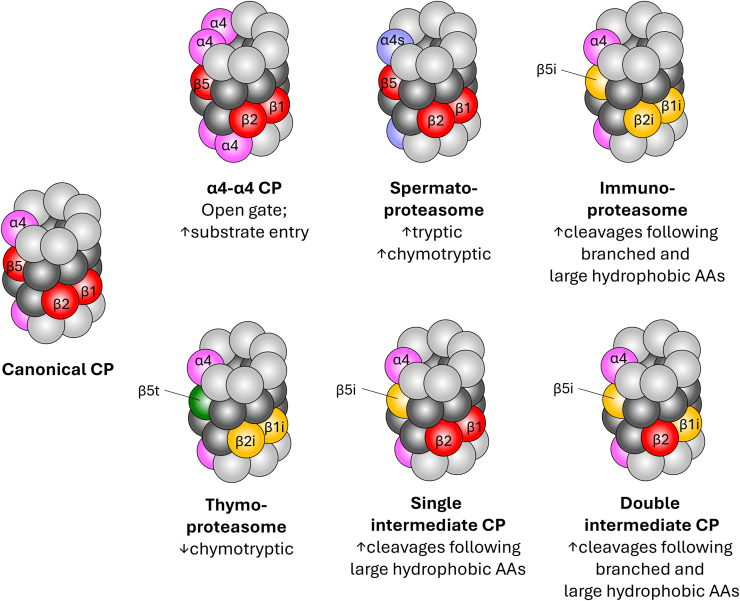
**Canonical and noncanonical core particles (CPs).** Known forms of the 20S CP are shown in *cartoon mode*. Coloring of CP subunits is as in Figure 1, with the following differences: α4 subunits are shown in *pink*; α4s is shown in *lavender*; immunoproteasome subunits β1i, β2i, and β5i are shown in *yellow*; and thymoproteasome subunit β5t is shown in *forest green*. Functional differences relative to the canonical CP are listed under each noncanonical CP.

Eukaryotic CPs have undergone explosive subunit diversification compared with those of bacteria and archaea (Fig. 2). All known eukaryotic CPs consist of heteroheptameric α and β rings that contain six to seven distinct α subunits and seven distinct β subunits, with the α and β rings of canonical eukaryotic CPs consisting of α1–α7 and β1–β7 subunits, respectively. Whereas bacterial and archaeal β rings typically contain a single β subunit and thus have 14 peptidase active sites per CP (39, 40), only three of the seven β subunits within eukaryotic β rings contain peptidase activity, such that eukaryotic CPs have only six total peptidase sites. This reduction in peptidase sites, however, is accompanied by a diversification of cleavage preferences; whereas bacterial and archaeal CPs tend to cleave substrates only after hydrophobic residues (41, 42), eukaryotic CPs can cleave after acidic, basic, or hydrophobic residues *via* the activities of the β1, β2, and β5 subunits, respectively. Thus, a broader array of peptide products is generated from eukaryotic *versus* simpler bacterial and archaeal CPs.

Most eukaryotes studied to date express seven or eight distinct α subunits; all species examined encode α1–α7. A subset of higher eukaryotes expresses an alternative form of α4 called α4s, which is encoded by a distinct gene (*PSMA8* in humans) (43). However, some invertebrates and plants express significantly higher numbers of α subunits. For example, most *Drosophila* species encode 11 (or more) α subunits (44), as does *Arabidopsis thaliana* (45). In most cases, the expression or incorporation of these individual α subunits into CPs has not yet been carefully examined.

#### CPs with noncanonical α rings

Two alternative forms of the CP arising from substitutions of subunits within the α ring have been well characterized to date. In the first, substitution of the α3 subunit with a second copy of α4 yields so-called α4–α4 CPs (46). Evidence for the formation of α4–α4 CPs in yeast and humans derives primarily from experiments where α4 was overexpressed or where α3 expression was suppressed (46, 47, 48); however, α4 is often overproduced in cancers, and elevation of α4 expression as little as twofold is sufficient to yield detectable α4–α4 CP formation in yeast (47). Interestingly, in both humans and in yeast, enhanced formation of α4–α4 CPs is associated with resistance to cadmium, which causes oxidative unfolding of proteins. Because the N terminus of α3 forms a key portion of the CP gate, its substitution with α4 yields a constitutively open gate (49) that may facilitate degradation of oxidized or unstructured proteins without the need for a regulator to facilitate access to the proteolytic sites.

Substitution of the α4 subunit with α4s yields CPs with the second known alternative form of the α ring, called spermatoproteasomes (sCPs). The α4s subunit appears to have emerged within the amniote lineage of Eukarya through duplication and specialization of the canonical α4 subunit, and is expressed only in spermatocytes, spermatids, and possibly in the ovary (43, 50). sCPs are essential for spermatogenesis, likely because of a role in degrading acetylated histones located at the sites of recombination-associated double-strand DNA breaks during meiosis I (51, 52). sCPs comprise as much as 92% of the total CP population in spermatocytes and spermatids; presumably due to a shift in expression from primarily α4 to primarily α4s rather than preferential incorporation of α4s over α4 (50). Interestingly, human α4 and α4s share ∼84% sequence identity and ∼94% similarity, with electrostatic differences in a solvent-facing helix and loop region being the major difference between the two. Thus, although the functional significance of this difference remains unknown, it is tempting to speculate that sCPs may participate in unique protein–protein interactions important for meiosis that are mediated *via* the solvent-exposed face of α4s and/or differential interactions with CP regulator(s). Consistent with this possibility, sCPs preferentially associate with the 19S RP in spermatocytes (50).

#### CPs with noncanonical β rings

Analogous to sCPs, alternative CPs can be formed by the substitution of catalytic β subunits. Several specialized forms of the three canonical catalytic β subunits emerged around the same time as the adaptive immune system evolved in jawed vertebrates (53). Consistent with this evolutionary timeline, the two best studied CPs harboring noncanonical β rings—the immunoproteasome (iCP) and the thymoproteasome (tCP)—each contribute to adaptive immunity. The iCP is constitutively expressed in hematopoietic and immune cells and is induced in other somatic cells in response to inflammatory cytokines (54, 55). The iCP contains substitutions of the canonical β1, β2, and β5 subunits with β1i, β2i, and β5i subunits, encoded by the *PSMB9*, *PSMB10*, and *PSMB8* genes, respectively. iCPs are responsible for generating antigenic peptides for presentation on major histocompatibility type I complexes. Substitution of β1 with β1i enhances cleavage of peptides harboring branched-chain hydrophobic amino acids at the P1 position (the residue on the N-terminal side of the scissile bond) (56). Similarly, β5i substitution yields a larger hydrophobic cavity in the S1 site that engages the P1 residue compared with the canonical β5 catalytic site, promoting more efficient cleavage after large hydrophobic side chains (56). Together, these alterations enhance the production of peptides harboring hydrophobic C-terminal residues, which are better loaded onto major histocompatibility type I complexes for antigen presentation.

The tCP occurs only in cortical thymic epithelial cells and contains β rings harboring the β1i, β2i, and β5t subunits in place of their canonical counterparts (57, 58). Whereas the impacts of β1i and β2i incorporation are thought to be the same as for the immunoproteasome, incorporation of β5t greatly reduces chymotryptic activity to produce peptide products depleted of hydrophobic C-terminal residues. The tCP is important for immune self-discrimination, as knockout of the *PSMB11* gene encoding β5t impairs CD8^+^ T-cell–positive selection (59, 60). The β5t subunit cleaves preferentially after hydrophilic residues present in a hydrophobic stretch (61); however, no molecular structures of the tCP currently exist, which has limited the understanding of how this preference is mediated by β5t substitution. Interestingly, the cortical thymic epithelial cells of β5t-deficient mice produce iCPs instead of tCPs (59), suggesting that β5t and β5i might be in direct competition for incorporation into nascent CPs in this tissue.

At present, no evidence supports the existence of CPs simultaneously harboring both noncanonical α rings and noncanonical β rings. However, hybrid or mosaic CPs containing mixtures of canonical and noncanonical β subunits have been identified (29); these CPs contain either one (β5i) or two (β1i, β5i) substitutions in the β ring and are called single- and double-intermediate CPs (siCPs and diCPs), respectively. Their production may occur transiently as cells induced by cytokines switch from the production of canonical CPs to iCPs and promote the production of unique antigens (29). Whether siCPs and diCPs have broader biological roles remains unknown. However, preliminary evidence suggests that they may have enhanced degradation of oxidized proteins (30).

### Plasticity between and within regulatory caps

As observed for the CP, the regulatory caps of actinobacteria and archaea tend to be fewer and simpler than in eukaryotes. These organisms typically utilize a single homohexameric ring of AAA family ATPases that caps the barrel ends of the CP and serves as the unfoldase motor for the CP. The best-characterized examples in bacteria are ARC of *R. erythropolis* (62) and Mpa of *Mycobacterium tuberculosis* (63), whereas the best-studied in archaea are the proteasome-activating nucleotidase of *Methanocaldococcus janaschii* (64) and the archaeal Cdc48 of *Thermoplasma acidophilum* (65, 66, 67). In each case, the ATPase ring directly captures substrates for degradation rather than relying on dedicated substrate receptors. Whereas actinobacteria encode only a single CP regulator, some archaea may encode as many as five regulators, including distinct forms of proteasome-activating nucleotidase and Cdc48, and a more distantly related AAA ATPase called AMA (66). In virtually all cases, the physiological roles of distinct archaeal regulators are unknown.

The composition of the canonical eukaryotic CP regulator, the 19S RP, is largely invariant. However, some parasites encode reduced RPs lacking the base subunit ubiquitin receptor Rpn13 and the lid subunits Rpn12 and DSS1 (68, 69, 70). At present, it is unclear how this impacts substrate recognition and degradation. Similarly, plants can encode multiple variants of most RP subunits (45), with some isoforms being selectively upregulated in response to various stresses. This suggests that distinct RPs may be formed in different plant tissues and/or under different circumstances. However, eukaryotic regulator plasticity generally comes in the form of alternative regulators that can dock onto the barrel ends of CPs to yield proteasomes with distinct substrate preferences and cleavage specificities. Interestingly, there may be some preference of specific regulators for specific variants of the CP (50, 71, 72), providing an additional layer of complexity to proteolysis whose underpinnings are not yet carefully explored.

**Figure 3:**
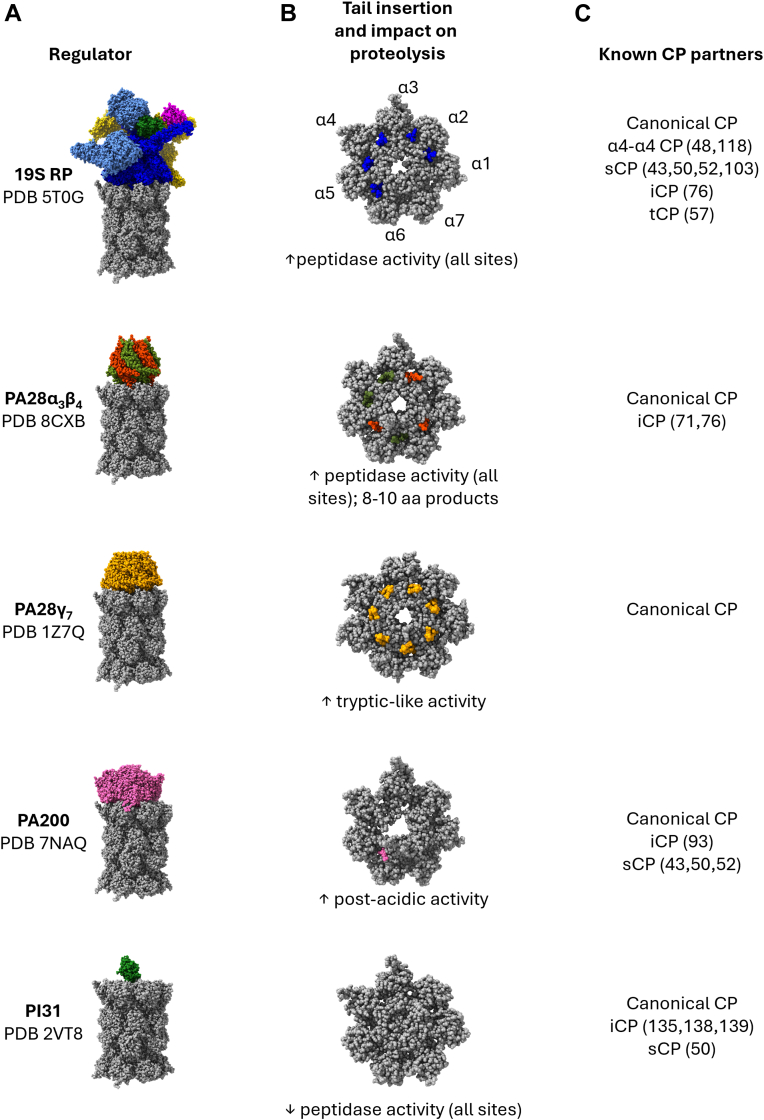
**Diversity among core particle (CP) regulators.***A*, known regulators of the 20S CP that interact with its barrel ends are shown. The corresponding Protein Data Bank files for each regulator–CP complex are listed. The structure of PA28γ is that of the trypanosome ortholog PA26 bound to the yeast 20S CP, whereas all other structures show human proteins. For PI31, the FP domain was modeled over the central pore of the CP (Protein Data Bank ID: 8QYO) in the absence of an available structure of this complex. *B*, interaction of the last seven amino acids of each docked regulator’s C termini with CP α ring pockets of a canonical CP is shown. The reported impact(s) of each regulator on CP peptidase activity are reported below. The impact of PA200 on CP activity in cells is controversial. *C*, CP variants reported to interact with each regulator are shown. References cited in this review that provide direct or reliable evidence of interaction with noncanonical CPs are shown in parentheses. FP, Fbxo7/PI31.

Of the noncanonical regulators, one CP inhibitor called PI31 (PSMF1; Fub1 in budding yeast) and three CP activators that modulate CP function are known (Fig. 3). The activators consist of two related heptameric regulators called PA28αβ (PSME1/2) and PA28γ (PSME3) as well as an unrelated monomeric regulator, PA200 (PSME4; Blm10 in yeast). None of these regulators harbors ATPase activity necessary for substrate unfolding (73, 74). They are thus proposed to be involved in the processing of intrinsically unstructured proteins (43, 74) or in promoting multiple cleavages in substrates by CPs harboring an RP on their opposing barrel ends (75, 76, 77). There are, however, some universal features shared by both the 19S RP and these three noncanonical regulators. All these activating regulators: (i) dock onto the barrel ends of the CP (Fig. 3*A*); (ii) insert their C termini into α-ring pockets to associate with the CP; and (iii) open the proteinaceous gate into the CP peptidase sites to promote substrate entry. Interestingly, the α-ring pockets into which each regulator docks, as well as their impacts on CP proteolysis, differ significantly (Fig. 3*B*).

#### PA28αβ and PA28γ

The PA28 family of regulators, also known as the 11S or REG proteins, forms a ring-shaped heptameric structure with unique roles in adaptive immunity and nuclear processes. PA28αβ is comprised of two proteins, PA28α and PA28β, and forms a heteroheptameric ring arranged α–β–α–β–α–β–β (78, 79). In contrast, PA28γ forms a homoheptameric ring composed of seven identical subunits and is likely the ancestral form of PA28. Whereas PA28αβ is induced by cytokines and functions in adaptive immunity similarly to the immunoproteasome subunits (80, 81), PA28γ is constitutively expressed in many cell types, is predominantly nuclear in localization (82), and has a less well-defined role in cellular physiology. It is clear, however, that both PA28αβ and PA28γ promote gate opening of the CP and alter the peptidase activities of the CP (83).

Biochemically, PA28γ preferentially stimulates the trypsin-like activity of β2 (84). Given its nuclear localization, this enhancement of post-K/R cleavage has been suggested to be important for the degradation of DNA-binding proteins, which are often enriched for these amino acids. In contrast, PA28αβ causes a less selective enhancement of peptidase activities, with the strongest effects on the chymotryptic-like and tryptic-like activities of β5 and β2. PA28αβ also reduces the size of peptide products produced by CPs, yielding peptides that are of appropriate size for antigen presentation (77). Consistent with this observation, PA28αβ appears to have emerged around the same time as the immunoproteasome subunits (85), is selectively enriched as regulatory caps on iCPs containing immunoproteasome subunits (Fig. 3*C*), and has seemingly been lost during evolution alongside iCP and tCP subunits in birds (85, 86, 87).

#### PA200/Blm10

PA200 is a unique monomeric regulator conserved from plants to yeast to humans and appears to be primarily involved in nuclear proteasome functions (43). PA200 is a ∼240 kDa protein composed almost entirely of alpha-helical HEAT repeats that form a dome-like structure over the CP (Fig. 3) (88, 89, 90, 91). Similar to the 19S RP, PA200 interacts with the CP *via* insertion of a conserved C-terminal HbYX motif into the α5–α6 pocket and appears to preferentially associate with open-gate CPs (92). Interestingly, the dome-shaped PA200 structure has small window-shaped openings through which unstructured substrates have been proposed to enter. The precise role of PA200 in proteasomal proteolysis is unclear; PA200 caps ∼10% of sCPs (50) (Fig. 3*C*) and was proposed to promote degradation of acetylated histones by PA200–CP complexes (43). Indeed, the C-terminal region of PA200 contains a domain similar to the acetyllysine-binding bromodomain. More recently, PA200 was reported to associate with iCPs in lung cancers, where it attenuated iCP activity and restricted antigenic diversity (93). However, PA200 is known to bind both to mature CPs as well as to CP assembly intermediates (94, 95, 96), which has led to proposals that it serves as a CP assembly chaperone or as a CP-specific karyopherin (97, 98). The yeast ortholog, Blm10, is important for regulating nuclear egress of proteasomes upon nutrient deprivation (98, 99), consistent with a role in regulating proteasome subcellular location.

#### PI31

PI31 is conserved from yeasts to humans and is both the sole known CP negative regulator cap and the sole known regulator that does not insert its C terminus into α ring pockets (Fig. 3). PI31 comprises a globular N-terminal Fbxo7/PI31 (FP) domain and a proline/glycine-rich unstructured C terminus. Several recent studies have clearly documented how the PI31 C terminus inhibits the CP (49, 70, 100). Contrasting other regulators, PI31 uses its C terminus and C-terminal domain to inhibit the CP active sites. The exact mode of inhibition varies somewhat between species, but generally, PI31 inserts through the CP pore and protrudes into the active sites of all three catalytic subunits; insertion of one PI31 molecule through each of the two CP pores yields complete inhibition of all six peptidase sites. The β1, β2, and β5 active sites are inhibited by a combination of hydrogen bonding with key catalytic residues to suppress their functions by the positioning of nonpermissive residues such as proline at the P_1_’ position of what would be the scissile bond, and in mammals, by winding through the active site in reverse orientation (*e.g.*, C terminal to N terminal) to mispresent what would be the scissile bond.

PI31 homodimerizes *via* its FP domain (100, 101, 102); however, any role of this domain in proteasome regulation is unknown. Positioning of the C terminus in the CP active sites is anticipated to pull the FP domain of PI31 close against the docking surface of the CP (Fig. 3*A*), which likely further suppresses proteasome activity by blocking binding of activating regulators. Consistent with this, CPs purified in the absence of ATP from *Vairimorpha necatrix*, an obligate intracellular parasite from the phylum *Microspora*, lack obvious activating regulators but contain densities consistent with PI31 (70). Interestingly, inclusion of ATP in the purification buffer yielded CPs lacking PI31 densities but capped by 19S RP. Thus, occupation of a CP surface by PI31 or activators is likely to be mutually exclusive and potentially regulated by the ATP status of the RP.

### How many forms of the proteasome exist?

Considering there are five known regulators and seven confirmed variants of the CP in humans, and each of the latter can bind a regulator on one or both ends, more than 100 unique proteasome variants could theoretically be formed. However, current evidence suggests that only a subset of these occur *in vivo* (Fig. 3*C*). Generally, the mechanisms that restrict the diversity of proteasome variants are poorly understood, but there appears to be some preference between particular CP variants and regulators. Generally, although canonical CPs have been described to associate with all five regulators and iCPs are enriched with PA28αβ, potential regulator preferences for noncanonical CPs, especially siCPs, diCPs, and α4–α4 CPs, are not yet established.

How the distribution of distinct regulators onto particular CPs is controlled has only recently been scrutinized. It is likely that a combination of tissue-specific subunit expression, cell type–specific CP and regulator abundances, and sequence- and conformation-specific differences in the surfaces of CP variants biases the distribution of CP–regulator pairs observed. In support of this, variations exist in the α ring surfaces of noncanonical CPs that may bias the association of particular CP variants with particular regulators (79, 103). However, in most cases, the affinities of particular CP variants for specific regulators have not been explored.

## How are proteasomes assembled?

The simpler, homomeric proteasomes of bacteria and archaea can assemble spontaneously without exogenous assistance. Enzymatically active bacterial (35, 62, 63, 104) and archaeal (64, 105) CPs and regulators can be recovered when their respective subunits are expressed in *Escherichia coli*. In contrast, heteromeric eukaryotic proteasomes are prone to misassembly, with a variety of misassembly products reported (106, 107, 108, 109, 110, 111, 112). To overcome this challenge, eukaryotic proteasomes rely both on intrinsic features of their subunits to help guide specific assembly steps, as well as exogenous, dedicated assembly chaperones that reinforce key intermediates and restrict nonproductive assembly events. Over the past ∼20 years, significant progress has been made in deciphering the assembly of both the canonical 26S proteasome and several noncanonical variants, with a barrage of recent structural studies of canonical CP assembly intermediates revealing the process in near-atomic resolution. Although similar structural studies for the assembly of noncanonical CPs have not yet occurred, it is likely they will help to further clarify the conserved and divergent features of canonical and noncanonical CP biogenesis and reveal the mechanisms that regulate the overall diversity of regulator–CP complexes that form *in vivo*.

### Canonical CP biogenesis

Assembly of canonical eukaryotic CPs, and likely all noncanonical CPs, begins with assembly of seven α subunits to produce a full α ring. This α ring then serves as a template for stepwise association of β subunits to yield half-proteasomes. Two half-proteasomes then associate to yield a preholoproteasome (PHP). The catalytic β subunits β1, β2, and β5 and their isoforms, as well as the noncatalytic subunits β6 and β7, are translated with N-terminal propeptides. These propeptides (i) prevent inactivating cotranslational acetylation of the catalytic N-terminal threonines (113, 114); (ii) help recruit neighboring subunits during assembly (94, 95, 113, 115); and most importantly, (iii) restrict the peptidase activity of β1, β2, and β5 subunits until completion of CP biogenesis. Formation of the PHP triggers the autocatalytic cleavage of propeptides present on these subunits, exposing the β1, β2, and β5 N-terminal catalytic threonines to yield proteolytically active CPs.

CP biogenesis occurs independently of the 19S RP or other CP regulators but is greatly facilitated by three dedicated assembly chaperones, PAC1–2 (Pba1–2 in yeast), PAC3–4 (Pba3–4 in yeast), and POMP (Ump1 in yeast) (46, 116, 117, 118, 119, 120). Chaperones PAC1–4 are primarily involved in α ring assembly, whereas POMP facilitates β ring assembly. Each of these chaperones associates with specific CP assembly intermediates and facilitates defined assembly events, and either dissociates or is destroyed upon CP maturation. CP assembly chaperones exert three major functions. They (i) stabilize fragile assembly intermediates formed *en route* to mature CPs; (ii) enforce ordered addition of CP subunits during assembly; and (iii) restrict nonproductive assembly events that would lead to dead-end misassembly products.

**Figure 4:**
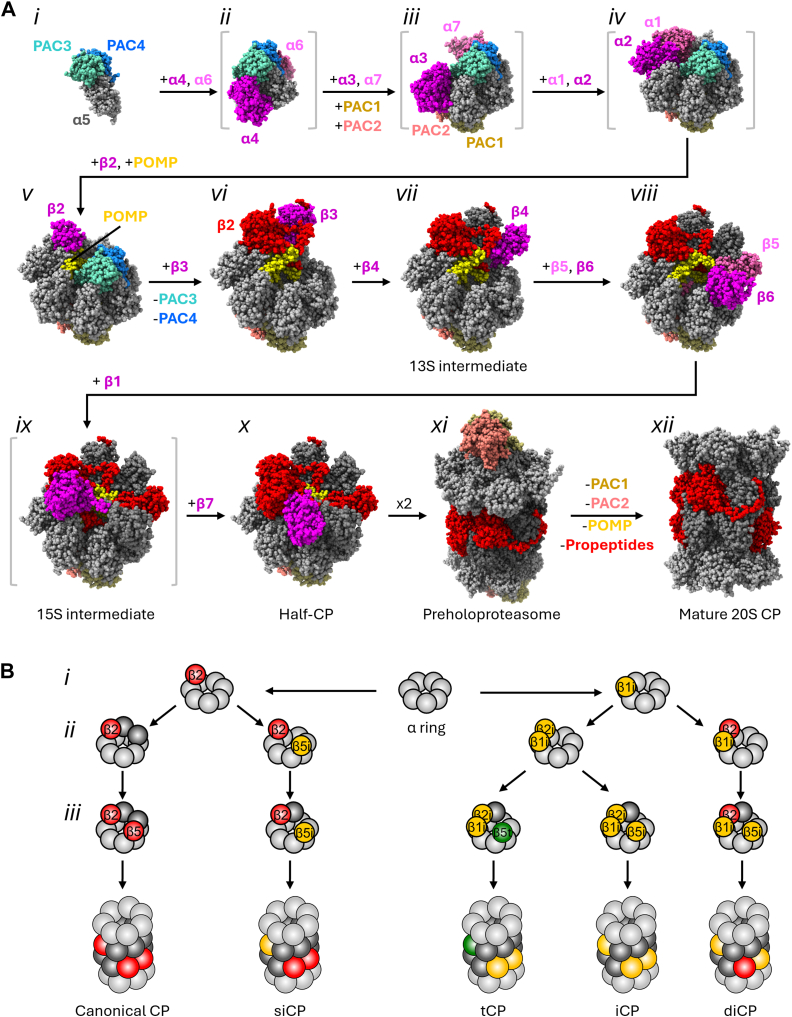
**Assembly of canonical and selected noncanonical core particles (CPs).***A*, structural snapshots of canonical CP biogenesis, derived from Protein Data Bank IDs 2Z5C (intermediate *i*), 8QYJ (intermediates *ii* to *v*), 8QYL (*vi*), 8QYM (*vii*), 8QZ9 (*viii*), 8QYN (*ix*, *x*), 8QYS (*xi*), and 8QYO (*xii*). For each stage, newly entered CP subunits are shown in *magenta* or *pink*, and they are faded to *light gray*, *dark gray*, or *red* for α, noncatalytic β, or catalytic β subunits, respectively. Complexes whose existence is supported biochemically but for which high-resolution structures are not currently available are shown in *gray brackets* and were prepared by omitting the relevant subunits from the indicated structure, which was otherwise closest in composition. For PAC1–PAC2, the stage of association is not known. However, we favor incorporation at stage *iii*, as this intermediate harbors both α pockets into which the C termini of PAC1 and PAC2 insert and permits formation of most of the buried surface area between this chaperone and the α ring. *B*, β subunit insertion order as a determinant of CP composition. Coloring is as in Figure 2. For clarity, assembly chaperones and multiple intermediate assembly steps have been omitted. Docking of β2 or β1i is proposed to serve as a commitment step to two separate linages of CPs (*i*). Addition of β5i prior to the β4-dependent incorporation of canonical β5 would then promote formation of siCPs over canonical CPs (*ii*, *left side*). In β1i-containing precursors, incorporation of β2 would promote formation of diCPs (*ii*, *right side*), whereas incorporation of β2i would yield formation of iCPs or tCPs upon incorporation of β5i or β5t, respectively. diCP, double-intermediate CP; iCP, immunoproteasome; siCP, single-intermediate CP; tCP, thymoproteasome.

At present, α ring formation is the least-understood part of CP biogenesis. Recombinant yeast subunit coexpression experiments have yielded α5+PAC3–4 (Fig. 4, complex *i*) and α4–5–6+PAC3–4 complexes (Fig. 4*ii*) (110, 118, 119), suggesting that α5–PAC3–4 may represent the nucleus of the α ring. PAC3–4 serves at least three main functions: first, its interaction with α5 blocks nonproductive, off-pathway association of α2 with α5 (110). This function is also supported by genetic data demonstrating that overexpression of α1 suppressed noncanonical CP assembly upon *PBA3* deletion in yeast (47), presumably by sequestering α2 that would otherwise mispair with α5. Second, PAC3–4 occupies a position on the interior surface of the α ring that, in mature proteasomes, would overlap with β subunits β3 through β7 (119, 121), and indeed, PAC3–4 is absent from intermediates containing β3–β7 (Fig. 4*vi–x*). This suggests that PAC3–4 prevents premature assembly of β subunits onto an incompletely assembled α ring. Third, by virtue of contact with multiple α subunits, PAC3–4 likely functions to stabilize the assembling α ring. In a structure of a full α ring bound by β2, PAC1–4, and POMP (Fig. 4*v*), PAC3–4 directly contacts α3 through α7, suggesting it may facilitate the insertion of α3 and α7 (Fig. 4*iii*) (121). Together, this evidence suggests that α ring formation likely initiates from an α5–PAC3–4 complex, with PAC3–4 functioning to stabilize α3–α7 as they add stepwise to the growing ends of the ring.

A second chaperone complex, PAC1–2, binds to the regulator-facing surface of the α ring. Docking of PAC1–2 is mediated in part by insertion of HbYX- and HbYX-like motifs at the C termini of PAC1 and PAC2 into the α5–α6 and α6–α7 pockets, respectively. PAC1–2 contacts all α subunits except α3, suggesting that it may help to scaffold α1 and α2 incorporation. The position of PAC1–2 on the outer α ring surface also prevents premature binding of proteasomal regulators that could interfere with subsequent assembly steps. Interestingly, PAC1–2 interacts directly with the N termini of multiple α subunits and induces an open gate–like conformation, but by virtue of inserting the PAC1 N terminus into the α ring pore, it occludes substrate entry (122).

Completion of α ring assembly initiates recruitment of β subunits to the assembling CP (Fig. 4*v-x*). POMP, an intrinsically disordered assembly chaperone, has been observed in an intermediate containing a full α ring and β2, in addition to PAC1–4 (Fig. 4*v*). It is not currently known whether β2 and POMP are corecruited; however, a proposed intermediate containing a full α ring bound by the yeast PA200 ortholog, Blm10, as well as β2 but without POMP ortholog, Ump1, has been recently reported (96), suggesting that β2 may incorporate first. Regardless, POMP together with PAC3–4 helps to stabilize β2 on the completed α ring to initiate β ring assembly. Incorporation of β2 is followed by recruitment of β3 (123). The β2 propeptide and a conserved β2 C-terminal extension (Fig. 4*vi*) form a pincer that, together with POMP, stabilizes incorporated β3. In this pincer arrangement, a region of POMP is observed contacting α4 in a manner that would sterically conflict with PAC3–4, suggesting either that recruitment of β3 triggers release of PAC3–4, or instead, that release of PAC3–4 permits incorporation of β3. Subsequent recruitment of β4 yields the well-characterized 13S assembly intermediate (Fig. 4*vii*), which has been observed both in yeast (95) and in human cells (121, 123).

Sequential addition of β5 and β6 (Fig. 4*viii*), followed by β1, occurs next based on the results of siRNA knockdown experiments (123), yielding the 15S intermediate (Fig. 4*ix*). Notably, complexes containing β1 but lacking β5 and β6 have been reported both for the canonical CP in β5 knockdown cells (123) and also for the iCP (124), suggesting that incorporation of β1 and β5/β6 may be stochastic rather than ordered. Docking of β7 completes the half-CP (Fig. 4, *x*) and licenses it for dimerization to yield a PHP (Fig. 4, *xi*). Incorporation of β6 and β7 is likely facilitated by the β5 propeptide, which is essential (or nearly so) for viability in yeast (115, 125). The propeptide makes extensive contact with the interior surfaces of these subunits in recent cryo-EM structures of the PHP (121, 126), and mutation of a key residue in the β5 propeptide contacting β6 yields a strong growth defect. Further, overproduction of β6 or β7 suppresses the lethality of β5 propeptide deletion (125).

PHP formation relies on a long C-terminal extension of β7 and on interfacial residues on the exposed β ring surfaces of the half-CPs that mate with one another. The β7 extension reaches across to the neighboring half-CP during PHP formation and inserts into a crevice between the exterior surfaces of β1 and β2 to stabilize the nascent PHP (94, 95). Upon PHP formation, the propeptides of catalytic subunits β1, β2, and β5 as well as noncatalytic subunits β6 and β7 are autocatalytically cleaved. In yeast, PHP formation causes a disordered-to-ordered transition in the C termini of β3 and β6 *via* formation of a salt bridge between the conserved terminal aspartate of these subunits and a conserved arginine within β5 and β2, respectively (125, 127, 128). Although the salt-bridging arginine is not directly involved in catalysis, it is almost directly adjacent to a second aspartate involved in catalysis, and thus, formation of this salt bridge likely repositions this catalytic residue to initiate propeptide cleavage and active site maturation. A similar structural repositioning of the catalytic residues has been observed in a cryo-EM structure of human PHPs expressed from insect cells (121) and likely underlies activation of β1 as well. A series of cryo-EM structures from yeast have helped support an order to these late events previously suggested by mutagenesis (114): the propeptide of β2 was visualized in the CP proteolytic chamber, but cleaved from β2, suggesting β2 matures first (128). In this structure, the propeptides of β6 and β7 are also invisible, suggesting they are cleaved by the newly activated β2, likely in *trans* across the opposite half-CP. Docking of the β7 C terminus with the opposing half-CP appeared to reorient the β1 active site for catalysis, suggesting it closely follows β2. Subsequent tightening of the half-CP–half-CP interface would in turn lead to β5 activation.

Processing of β subunit propeptides is functionally coupled to the release of PAC1–2 and the destruction of POMP to yield a mature 20S CP (Fig. 4*xii*). Formation of the PHP traps POMP inside the central CP proteolytic cavity, so it must be destroyed to clear the catalytic chamber (116). Interestingly, POMP and the β5 propeptide make direct contact with PAC1 from the PHP interior, and this contact is presumably lost upon POMP destruction and β5 maturation. Loss of these avid interactions with PAC1 is proposed, along with remodeling of the α ring, to promote PAC1–2 dissociation (128, 129, 130). Whether the processing of POMP and the release of PAC1–2 are coordinated between the two CP halves is not currently known. However, PHPs bound by PAC1–2 at both (122) or only one (121) end have been observed by cryo-EM, raising the possibility that POMP degradation and PAC1–2 release by the two half-CPs are not directly coupled.

### Assembly of noncanonical CPs

#### α4–α4 CPs

The α3 subunit is the sole nonessential CP subunit in budding yeast (46), which provided the first hint of plasticity within the composition of the CP. Considering that α4–α4 CPs differ from canonical CPs only by a single subunit, it is likely that most of the assembly mechanisms observed for canonical CPs also participate in α4–α4 CP assembly. In yeast and in human cells, PAC3 or PAC4 deficiency results in the formation of a mixture of canonical and α4–α4 proteasomes, implicating PAC3–4 as a negative regulator of α4–α4 CP formation (46, 48, 118). Recent structural studies have provided some insight into this function. PAC3–4 makes direct contact with α3 in canonical CP assembly intermediates (Fig. 4, *iii*) and likely drives preferential incorporation of α3 adjacent to α4 *via* this discriminatory contact. It is therefore reasonable that under conditions of exceptionally rapid proteasome biosynthesis, the catalytic amounts of PAC3–4 available for assembly may become limiting, resulting in an increase in α4–α4 CP formation, although this has not yet been carefully tested.

Formation of α4–α4 proteasomes is also likely governed by the relative expression levels of α4. This is supported by the observation that doubling the expression of α4 in yeast yields detectable α4–α4 CPs (47), and that overproduction of α4 in human cells yields α4–α4 CPs in abundances proportional to α4 levels (48). In human cells, α4 levels are regulated by the tumor suppressor BRCA1, which ubiquitinates free α4 for degradation (131). Interestingly, phosphorylation of α4 by the oncogenic kinases ABL or ARG prevents BRCA1-dependent degradation of α4, suggesting that α4–α4 CPs may be enriched in certain human tumors with mutant BRCA1 or overproduction of ABL or ARG. Considering that cancers often experience elevated oxidative stress, formation of α4–α4 CPs may provide tumors with enhanced resistance to an oxidizing environment by virtue of their constitutively open gates (49) that render them better able to degrade oxidized or unstructured proteins (48, 118).

#### sCP assembly

As for α4–α4 CPs, α4s subunit abundance is likely the key regulator of sCP formation. Gonad-specific α4s expression would thus serve to restrict sCP formation to reproductive tissues. Three pieces of evidence support this supposition: first, α4s expression rises proportionally to the accumulation of sCPs during differentiation of spermatogonia into spermatids (50); second, expression of α4s in human embryonic kidney (HEK) 293T cells is sufficient to promote sCP formation (103); and finally, sCP formation in α4s-expressing HEK293T cells was further enhanced upon knockdown of α4. Further, this formation of sCPs in a nontestes cell type implies that no testis-specific assembly factors are likely to exist. Quantitative proteomics has identified substantially less POMP present in α4s immunoprecipitates compared with α4 immunoprecipitates (50), indicating that POMP may be less important for the formation of sCPs compared with canonical CPs.

#### iCP and tCP biogenesis

As is the case for α4–α4 and sCPs, knowledge of iCP and tCP biogenesis has lagged that of canonical CPs and remains hampered because of a lack of available structures of mature tCPs or iCP/tCP assembly intermediates to guide mechanistic studies. However, the available data indicates that, whereas α ring assembly likely proceeds identically to that for canonical CPs, there are several divergences in iCP/tCP β ring assembly. In immune cells and nonimmune cells exposed to interferon gamma (IFNγ), iCP formation is dominant over canonical CP formation (132). As for the canonical CP, incorporation of early β subunits is triggered by completion of the α ring. Although β2 incorporation initiates canonical CP β ring assembly, β1i is likely the first β subunit to incorporate into the β rings that will ultimately yield iCPs, tCPs, and diCPs (Fig. 4*Bi*) (133). This is supported by observations that knockdown of β3 in IFNγ-stimulated HeLa cells causes accumulation of a full α ring with β1i and β2i associated (134) (Fig. 4*Bii*), and the finding that β1i could incorporate into assembly intermediates without β2i in cultured mammalian cells (133), albeit at reduced efficiency. In contrast, β2i incorporation is fully dependent upon β1i incorporation (135). Notably, the propeptide of β5i appears important for the exclusion of canonical β1, β2, and β5 (136). Together, this implies that incorporation of β1i prior to canonical β2 may serve as a switch that toggles the PAC1–4-bound α ring assembly intermediate toward iCPs, tCPs, and diCPs (Fig. 4*Bi*).

A second peculiarity of iCPs and tCPs is that β5i or β5t incorporation appears independent of β4, as presumed assembly intermediates consisting of an α ring, β1i, β2i, β3, and β5i/β5t are observed when β4 is knocked down in IFNγ-stimulated HeLa cells (Fig. 4*Bii*) (137). The above-mentioned finding of an α ring + β1i, β2i, β3, and β4 in mouse cells thus suggests that β4 and β5i/β5t can incorporate in either order. Incorporation of β5i independent of β4 occurs even when the canonical β5 propeptide is transplanted onto β5i, or when β1i and β2i are simultaneously knocked down. In contrast, the β5t propeptide is dispensable for β4-independent incorporation (137). This independent incorporation of β5i/β5t thus may be important for ensuring the homogeneity of iCPs and tCPs by rendering increased opportunity for β5i/β5t incorporation compared with the β4-dependent incorporation of canonical β5 (Fig. 4*Biii*) and also is likely to be responsible for the formation of siCPs during upregulation of iCP biogenesis in cytokine-stimulated cells (Fig. 4*Biii*). Subsequent steps of iCP and tCP assembly appear to mirror that of canonical CPs, with sequential β6 and β7 incorporation.

It is likely that all known canonical CP chaperones participate in functionally equivalent roles during iCP and tCP biogenesis, based on their presence in various iCP and tCP assembly intermediates (137, 138). However, the CP regulator PI31 may additionally function as an iCP assembly factor *via* an unknown mechanism. Overexpression of PI31 has previously been observed to suppress maturation of the β1i, β2i, and β5i subunits and reduce the resultant presentation of iCP-dependent peptide epitopes by major histocompatibility I complexes (135). This finding was supported by CRISPR–Cas9-mediated deletion of PI31 in multiple cell types (138), which similarly caused accumulation of iCP precursor complexes most similar to the 13S and 15S intermediates but with no impact on canonical CP formation. Although PI31 clearly associates with mature canonical CPs and iCPs in purified systems, PI31 could not be detected bound to any iCP assembly intermediates, suggesting that it may associate with these intermediates very weakly or highly transiently. Intriguingly, whereas PI31 acts to suppress the activity of the canonical CP (Fig. 2), iCPs retain the ability to cleave the C-terminal region of PI31 that inserts into the proteolytic cavity (139), suggesting a possible β-subunit scaffolding function reminiscent of that of POMP. An appealing model is that PI31 may be fully destroyed or may undergo partial proteolysis upon completion of its assembly function, resulting in the release of its globular N-terminal FP domain from newly matured iCPs. Intriguingly, *Microsporidia* have highly reduced genomes and lack an obvious POMP ortholog, but they have retained PI31. Whether PI31 is required for CP assembly in a POMP-like role in these reduced eukaryotes is a fascinating question that awaits investigation.

### Assembly of regulators

#### PA28 complexes

PA28αβ and PA28γ hold the capacity to self-assemble without exogenous assistance, as recombinant heteroheptameric PA28αβ complexes and homoheptameric PA28γ can be produced readily in *E. coli* (83). However, PA28α and PA28β can form homoheptamers not observed in living cells when heterologously expressed alone in *E. coli* (83, 140). Further, whereas PA28αβ complexes purified from human cells appear to exist as PA28α_3_β_4_ complexes arranged α–β–α–β–α–β–β (79), recombinant PA28αβ produced by coexpression yields PA28α_4_β_3_ complexes in the arrangement α–α–β–α–β–α–β (141). This has been attributed to a more thermodynamically favorable interface between α subunits (141). At present, the mechanism that may restrict the formation of PA28α homomers or non-natural PA28αβ complexes *in vivo* remains undiscovered. This, in principle, may be controlled *via* subunit expression stoichiometry, or alternatively, *via* one or more assembly chaperones that await discovery.

#### 19S RP lid assembly

Like eukaryotic CPs, the 19S RP relies on dedicated assembly chaperones and intrinsic features of its subunits to ensure rapid and efficient assembly (142). In contrast to the CP, very few structures of intact RP assembly intermediates have been solved, which has slowed progress in understanding the mechanistic determinants of RP formation. Most—but not all—evidence supports independent assembly of the lid and base subcomplexes, which subsequently associate with one another and with ubiquitin receptor Rpn10 to yield an intact, chaperone-bound 19S RP.

Lid assembly has been studied primarily using genetic experiments in yeast and biochemical reconstitution experiments in *E. coli*. In contrast to the base and CP, it assembles without help from dedicated chaperones (143, 144, 145, 146). Although lateral interactions between winged helix domains of Rpn3, Rpn5-7, Rpn9, and Rpn12 contribute to lid assembly, the process is guided primarily by the formation of a helical bundle comprised of the C termini of all lid subunits except DSS1 (Fig. 1) (143). The formation of avid interactions within this helical bundle upon successive subunit docking thereby enforces a hierarchical order to lid assembly (145, 146, 147, 148, 149).

**Figure 5:**
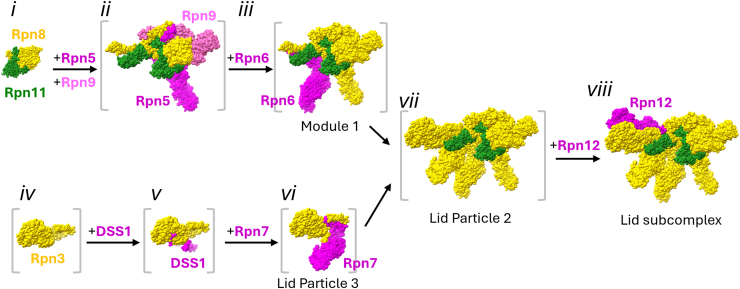
**Assembly of the RP lid subcomplex.***A*, structural snapshots of lid biogenesis, derived from Protein Data Banks 4O8X (yeast Rpn8–Rpn11 heterodimer lacking C-terminal helices, complex *i*) and 3JCK (yeast lid, complexes *ii*-*viii*). For each stage, newly entered lid subunits are shown in *magenta*, and they are faded to *yellow* in subsequent stages. Complexes whose existence is supported biochemically but for which high-resolution structures are not currently available are shown in *gray brackets* and were prepared by omitting the relevant subunits from Protein Data Bank 3JCK. RP, regulatory particle.

In both yeast and in human cells, lid biogenesis occurs through the parallel assembly of two complementary intermediates. The first intermediate consists of subunits Rpn5, Rpn6, Rpn8, Rpn9, and Rpn11 and is often referred to as Module 1 (Fig. 5*iii*) (150). In yeast, Module 1 assembly initiates with the association of Rpn8 and Rpn11 to yield a heterodimeric deubiquitinase unit (Fig. 5*i*). The interaction between these two proteins is mediated primarily by their globular Mov34/Pad1, N-terminal (MPN) domains (143, 151, 152). The isolated Rpn8–Rpn11 unit, while enzymatically active, is ∼500-fold less efficient than in mature 26S proteasomes due to poor ubiquitin affinity (151, 152). Rpn8 and Rpn11 contain the largest C-terminal helices among lid subunits and thus serve as the scaffold upon which the other subunits assemble. In support of this, the isolated C termini of Rpn8 and Rpn11 support formation of full lid-like complexes (143), and deletion of the Rpn8 or Rpn11 C terminus is sufficient to ablate lid formation (143, 153). Rpn5 and Rpn9 next associate with Rpn8 and Rpn11 *via* their respective C-terminal helices (Fig. 5*ii*), followed by incorporation of Rpn6 to yield Module 1 (Fig. 5*iii*). In knockdown experiments in human cells, a similar pathway has been observed that yields Module 1; however, Rpn11 appears to be less important for the formation of this module, as a nearly complete lid lacking only Rpn11 and Rpn12 accumulates upon Rpn11 knockdown (148).

The second lid intermediate is a trimeric complex consisting of subunits Rpn3, Rpn7, and DSS1 called Lid Particle 3 (LP3) (Fig. 5*iv-vi*). Although Rpn3 and Rpn7 are neighbors in the mature lid subcomplex, they do not interact stably in isolation (146). DSS1 is an intrinsically disordered protein that interacts with Rpn3 and Rpn7 *via* two conserved acidic patches separated by a flexible region. In yeast, DSS1 serves as a tether to secure these two proteins to each other until their interaction can be reinforced by docking of LP3 with Module 1, upon which this tethering function becomes dispensable (146). DSS1 may also facilitate the folding or stability of Rpn3, as the yield of yeast Rpn3 expressed in *E. coli* is improved by DSS1 coexpression (146).

Association of Module 1 and LP3 yields a nearly complete lid assembly intermediate, termed Lid Particle 2 (LP2) (Fig. 5*vii*), which lacks only Rpn12 (147, 148, 149). Although Rpn12 makes very little contact with the base subcomplex in the context of mature 26S proteasomes, its incorporation into the assembling lid is critical for docking of the lid to the base. Structural studies have suggested that LP2 may exist in a closed conformation in which the palm of the lid that would contact the base ATPase ring is obscured by the N-terminal finger of Rpn6 (Fig. 1) and/or the MPN domains of Rpn8 and Rpn11 (145, 154). This autoinhibited conformation is relieved upon Rpn12 incorporation, resulting in a mature and assembly-competent lid subcomplex. Relief of autoinhibition is mediated primarily by docking of the Rpn12 C terminus into the lid helical bundle formed by the C termini of most other lid subunits. Provision of a peptide corresponding to the Rpn12 C terminus both released the Rpn6 finger domain from the palm of the lid and promoted stable interaction between the lid and base using purified yeast components (145). Curiously, in a cryo-EM structure of the isolated lid subcomplex, the Rpn8–Rpn11 MPN domains remain seated in the palm where they would sterically interfere with binding of the base (154); whether the position of Rpn8–Rpn11 is dynamic in solution and can transiently adjust to accept the base, or whether there is instead a trigger or signal for repositioning of this deubiquitinating module is not yet clear.

#### 19S RP base assembly and docking to the lid

Similar to lid biogenesis, assembly of the base subcomplex occurs *via* the formation of multiple smaller intermediates in parallel that then associate with one another to complete assembly. However, in contrast to lid biogenesis, base assembly is heavily dependent upon four evolutionarily conserved assembly chaperones. These four chaperones, PAAF1, p28/gankyrin, p27, and S5b (Rpn14, Nas6, Nas2, and Hsm3 in yeast), each recognize the small domain of a particular Rpt ATPase subunit at the perimeter of the ATPase ring and serve distinct functions during base biogenesis. Atomic structures of each chaperone have been determined, in some cases bound to a fragment of their ATPase partner (155, 156, 157, 158, 159, 160). This has enabled modeling of most chaperones onto available structures of mature 26S proteasomes, providing much of the current insight into their functions.

**Figure 6:**
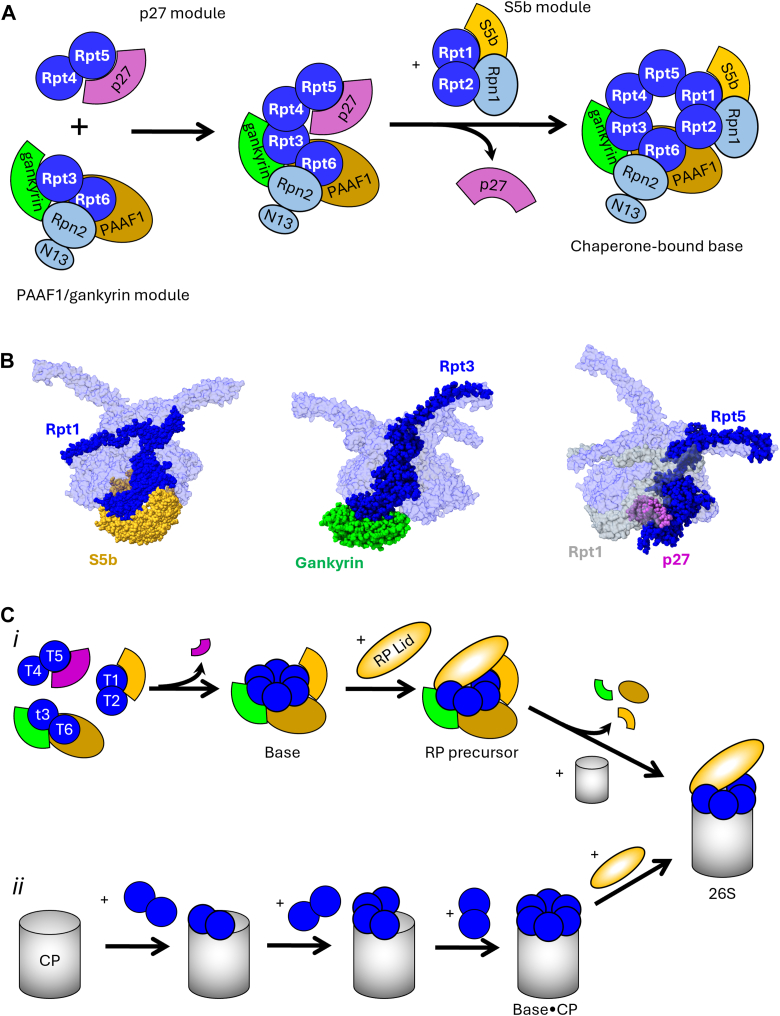
**Assembly of the base subcomplex and integration with the lid.***A*, schematic representation of the base subcomplex assembly pathway. Coloring of proteasome subunits is as in Figure 1, with base assembly chaperones PAAF1, gankyrin, p27, and S5b shown in *brown*, *green*, *purple*, and *goldenrod*, respectively. *B*, molecular modeling of S5b, gankyrin, and p27 onto the base ATPase ring. Yeast S5b (PDB ID: 3LVF), yeast gankyrin (PDB ID: 2DZN), and yeast p27 (PDB ID: 3WHL) in complex with the small domain of their cognate ATPase subunits were superimposed onto their Rpt binding partner (*dark blue*) within the 26S proteasome ATPase ring from PDB 6FVT. Rpt subunits not in direct contact with the chaperone are partially transparent. For the p27 structure, Rpt1 is shown in *gray* to illustrate steric conflict between p27 and the Rpt1–Rpt2 module that necessitates prior dissociation of p27 for docking of the S5b module, as shown in (*A*). *C*, a schematic model of RP formation and attachment to the CP (*i*) and an alternative model in which the CP scaffolds base assembly (*ii*). Base non-ATPase subunits and ubiquitin receptor Rpn10 are omitted for simplicity. For the CP-templated pathway, the ordered association of ATPase dimers as shown is hypothetical. CP, core particle; PDB, Protein Data Bank.

Base biogenesis proceeds through the formation of three modules: Rpt1–Rpt2–Rpn1–S5b, PAAF1–Rpt6–Rpt3–gankyrin, and Rpt4–Rpt5–p27 (161, 162, 163, 164, 165, 166). Association of these modules in a stepwise fashion in turn yields a fully assembled, chaperone-bound base subcomplex (Fig. 6*A*). Module formation initiates with the cotranslational heterodimerization of ATPase subunits Rpt1–Rpt2, Rpt3–Rpt6, and Rpt4–Rpt5 (167), followed by decoration of the ATPase dimers with non-ATPase subunits Rpn1, Rpn2, and Rpn13 and their cognate chaperones (161, 162, 163, 164, 165, 168). Whereas S5b acts as a scaffold for assembly of its module (159) (Fig. 6*B*, *left*), p27 is dispensable for Rpt4–Rpt5 pairing (162). Modeling suggests that gankyrin contacts only Rpt3 (Fig. 6*B*, *center*) and thus also is dispensable for Rpt3–Rpt6 pairing; the position of PAAF1 on Rpt6 has not yet been experimentally determined, and thus, a putative scaffolding role remains unknown.

Association of the three chaperone-bound modules occurs stepwise both in humans and in yeast, although the order may differ between species (15, 161). In yeast, a complex consisting of the PAAF1–gankyrin module and the p27 module has been observed *via* coimmunoprecipitation (15) and by coexpression in *E. coli* (169). Further, p27 has not been observed associated with a full ATPase ring. From these observations, it was proposed that p27 dissociates prior to completion of base assembly and that incorporation of the S5b module completes base assembly. This is supported by the structure of the α-helical N terminus of p27 bound to a fragment of Rpt5, which, when modeled onto a complete ATPase ring, reveals steric conflict between p27 and Rpt1 in the incoming S5b module (Fig. 6*B*, *right*) (170). Release of p27 has been proposed to be triggered in a manner dependent on both recruitment of the S5b module and on stimulation of Rpt4 ATPase activity (169), although the mechanistic details of this process remain uncertain. In human cells, knockdown of Rpt4 or Rpt5 instead resulted in accumulation of an RP-like species containing all lid and base subunits except Rpt4–Rpt5, suggesting that incorporation of the p27 module may complete base (or RP) assembly in humans (161).

Although the base shares a similar ring-like architecture with CPs, ATP binding and hydrolysis by Rpt subunits render them conformationally dynamic *versus* the comparatively rigid CP subunits. These conformational movements, if uncontrolled during assembly, could interfere with the successful meshing of adjacent subunits to one another (142). It is likely that ATPase activity is restricted within base intermediates, at least in part by one or more assembly chaperones. Indeed, base intermediates lack ATPase activity but become active upon their assembly into the RP (166), and the chaperone-bound base displays reduced ATPase activity compared with the chaperone-free base (171). Most evidence suggests that the completed lid and base subcomplexes associate with one another to yield an RP precursor bound by PAAF1, gankyrin, and S5b. Ubiquitin receptor Rpn10 likely associates with the RP precursor at this stage, as it has not been observed bound to the isolated lid or base (14). Cryo-EM structures of the gankyrin-bound RP from human cells suggest that the ATPase ring may be able to adopt an unusually open conformation in the isolated RP, whereby the Rpt3–Rpt6 heterodimer is splayed outward from the ATPase ring center and has substantially reduced contact with the neighboring Rpt4 and Rpt2 ATPases (172). However, any significance of this to RP biogenesis has not yet been established.

One of the most intriguing questions regarding RP assembly at present is how chaperone action is regulated during the assembly process. Formation of 26S proteasomes from the chaperone-bound RP and mature CPs requires disengagement of PAAF1, gankyrin, and S5b to relieve steric conflict between the chaperones and the CP (144, 163, 164, 173). At present, the mechanisms that control the orderly release of these chaperones from the nascent RP are unknown but are likely coupled to ATP hydrolysis (144, 173). The best evidence for this exists for gankyrin. Gankyrin interferes with binding of the base to the lid *in vitro* when the poorly hydrolyzed ATP analog ATPγS is provided and reciprocally inhibits binding of the base to the CP when ATP (which is presumably rapidly hydrolyzed to ADP + P_i_) is provided, suggesting that nucleotide-dependent conformational movements in the base exploit gankyrin to control the timing or order of lid, base, and CP association (144, 173). Modeling gankyrin onto mature 26S proteasomes indicates steric conflict with gankyrin that alternates between the lid and base during the ATPase cycle. This is likely to promote the release of gankyrin from the ATPase ring. In support of such a mechanism, disruption of conformation-specific contacts between lid subunits Rpn6 or Rpn5 and the base ATPase ring impairs 26S proteasome formation and causes accumulation of gankyrin-bound RP in a manner dependent on the nucleotide state of key ATPase subunits in the base (144, 173).

It is less clear how S5b and PAAF1 are released from assembly intermediates or nascent 26S proteasomes. Although S5b interacts with the ATPase ring similarly to gankyrin, it associates with Rpt1, which is located on the opposite side of the ATPase ring from the lid. S5b would thus likely rely on conflict with the CP or ATP-dependent rearrangements of the ATPase ring for release. Interestingly, a recent combined crosslinking-mass spectrometry and cryo-EM study yielded a model of S5b on the RP in which its C terminus inserted into the pore of the ATPase ring from the underside of the ring. This arrangement not only blocks CP interaction but also suggests that S5b may sense movements within the ATPase motor that disengages it from the RP upon stimulation of ATPase activity (174). Although the position of PAAF1 on base intermediates is uncertain, immunoprecipitation of PAAF1 from yeast cells enriches the base several fold over the RP (165), suggesting that it may slowly dissociate from the RP upon lid attachment.

### Plasticity in proteasome biogenesis—circuits in sequence or pathways in parallel?

With 66 individual protein subunits, there are nearly limitless ways in which a 26S proteasome could theoretically assemble. It is thus an astonishing feat of evolution that these enormous and complicated structures assemble rapidly and efficiently in cells. As described above, biogenesis of both CPs and RPs has largely been considered to occur *via* well-defined, inflexible paths, and most evidence available is certainly consistent with such dominant assembly pathways. However, the literature is peppered with reports of complexes with subunit compositions that differ from the intermediates described above (47, 110, 111, 112, 153, 160, 166, 175, 176, 177, 178). This raises an important unanswered question: are these peculiar species experimental artifacts, dis/misassembly products, or evidence of alternative assembly pathways? The explosion of structural studies of proteasomes and their intermediates over the past ∼15 years has transformed our understanding of this fascinating macromolecular machine and how it is assembled. Nonetheless, nearly all knowledge of proteasome biogenesis thus far is derived from static snapshots of metastable complexes. It remains formally possible that many extremely short-lived intermediates have been missed, or that other assembly pathways populated at comparatively low levels have thus far gone unrecognized. The literature hints that this is likely; we highlight two such cases below.

In contrast to the other CP regulators, PA200 has repeatedly been observed to be associated with incomplete 20S complexes in cells (92, 94, 95, 97). In yeast, PA200/Blm10 is primarily nuclear (99) and copurifies with POMP, suggesting it associates with CP assembly intermediates (97). In yeast, PA200 is important for nuclear localization of the CP (98), leading to the proposal that PA200 may act as a CP-specific karyopherin during assembly. However, recently, it was demonstrated that PA200 helps to stabilize PAC3–4-containing complexes in yeast when PAC1–2 are absent (179), and a recent high-resolution structure of the 13S assembly intermediate displayed PA200 associated with the outer face of the α-ring in a manner mutually exclusive with PAC1–2 (96), suggesting an alternative CP assembly pathway in which PA200 substitutes for one or more PAC1–2 functions.

Similarly, whereas chaperone-bound free base and chaperone-bound RP are readily visualized in normal yeast and human cells, several studies have suggested that the CP may act as a scaffold upon which the base or RP can be assembled (118, 153). In yeast, deletion of PAC3, PAC4, or POMP causes an accumulation of base assembly intermediates (118), suggesting a potential templating role for the CP surface. Similarly, yeast lacking all four base assembly chaperones are viable and can assemble intact 26S proteasomes, albeit with reduced abundance (162, 165). Taken together, these data suggest that chaperone-dependent and CP-templated base assembly may provide redundant pathways for base formation *in vivo* (Fig. 6*C*).

The use of the CP as a scaffold for assembly of the base would presumably yield base–CP complexes as an intermediate *en route* to 26S proteasomes. Formation of base–CP complexes has been demonstrated in experiments with purified components (160) as well as in yeast cells upon genetic repression of lid formation (153), suggesting such an intermediate can, in principle, form. Further, an incomplete lid subcomplex containing subunits Rpn5, Rpn6, Rpn8, Rpn9, and Rpn11 was detected, associated with base–CP complexes expressing mutant Rpn11 lacking its C terminus. Provision of the Rpn11 C terminus in *trans* promoted the assembly of mature 26S proteasomes, consistent with *de novo* lid assembly on existing base–CP complexes (153). Thus, the emerging question for each of these cases is whether these alternative assembly pathways are populated appreciably during normal proteasome synthesis in cells. In yeast, the abundances of the assembly chaperones are estimated to be on average ∼10-fold lower than the abundances of proteasome subunits (180). Further, it is well established that proteasome biosynthesis is upregulated to match the proteolytic load in response to various cellular stresses, whereas the expression of these chaperones largely remains constant (181). It is thus attractive to speculate that under some environmental conditions or cellular stresses, the catalytic amounts of chaperones may become limiting, resulting in the population of alternative assembly pathways such as those suggested above.

### Future directions

Nearly 50 years have elapsed since Fred Goldberg’s laboratory first reported an ATP-dependent machinery that degraded abnormal proteins (6), now known to have been the 26S proteasome. Despite years of study, our picture of how proteasomes are built rapidly and efficiently in cells is still in its infancy. Clarity has begun to emerge in terms of the architecture of proteasome intermediates along the major assembly pathways reviewed herein, but we still have an incomplete understanding of the structural mechanisms that mediate the formation of several noncanonical CPs. Further, although the pairing of regulators with CP variants appears to be regulated, the rules governing these pairings and how they may change in response to environmental cues remain very poorly delineated. Finally, whether alternative assembly pathways hinted at in the literature contribute appreciably to proteasome biogenesis in cells under varying environmental or cellular conditions warrants further investigation. The development of new experimental tools to quantitatively track assembly in time and space will likely reveal previously unappreciated facets of proteasome biogenesis that will keep proteasome researchers busy for years to come.

## Conflict of interest

The authors declare that they have no conflicts of interest with the contents of this article.
